# How Thymocyte Deletion in the Cortex May Curtail Antigen-Specific T-Regulatory Cell Development in the Medulla

**DOI:** 10.3389/fimmu.2022.892498

**Published:** 2022-05-25

**Authors:** Chenglong Wang, Stephen R. Daley

**Affiliations:** Centre for Immunology and Infection Control, School of Biomedical Sciences, Faculty of Health, Queensland University of Technology, Brisbane, QLD, Australia

**Keywords:** thymus, T-cell selection, T-cell tolerance, T-cell deletion, T-regulatory cells, self-antigen recognition, autoimmune disease

## Abstract

CD4^+^ T cell responses to self-antigens are pivotal for immunological self-tolerance. Activation of Foxp3^–^ T-conventional (T-conv) cells can precipitate autoimmune disease, whereas activation of Foxp3^+^ T-regulatory (T-reg) cells is essential to prevent autoimmune disease. This distinction indicates the importance of the thymus in controlling the differentiation of self-reactive CD4^+^ T cells. Thymocytes and thymic antigen-presenting cells (APC) depend on each other for normal maturation and differentiation. In this Hypothesis and Theory article, we propose this mutual dependence dictates which self-antigens induce T-reg cell development in the thymic medulla. We postulate self-reactive CD4^+^ CD8^–^ thymocytes deliver signals that stabilize and amplify the presentation of their cognate self-antigen by APC in the thymic medulla, thereby seeding a niche for the development of T-reg cells specific for the same self-antigen. By limiting the number of antigen-specific CD4^+^ thymocytes in the medulla, thymocyte deletion in the cortex may impede the formation of medullary T-reg niches containing certain self-antigens. Susceptibility to autoimmune disease may arise from cortical deletion creating a “hole” in the self-antigen repertoire recognized by T-reg cells.

## Introduction

Foxp3^+^ T-regulatory (T-reg) cells are an immunosuppressive lineage of T cells essential for immune tolerance (1). The development and function of T-reg cells depend on interactions between the T cell receptor (TCR) and peptide-major histocompatibility complex class II (pMHCII) antigens on the surface of other cells (2, 3). Some pMHCII self-antigens induce thymic lymphocytes (thymocytes) to upregulate Foxp3 (4–6); we refer to these pMHCII self-antigens as T_regitopes_ (T-reg epitopes). Some self-peptides fail to form a T_regitope_ because they cannot bind stably to the peptide-binding grooves of any MHCII alleles expressed in a given organism (7). In addition, the expression pattern of a self-peptide affects its capacity to form a T_regitope_. Self-peptides not expressed in the thymus do not affect the development of responding thymocytes, whereas highly expressed self-peptides induce thymocyte deletion (8, 9). Highly expressed self-peptides induce thymocyte deletion because the high number of pMHCII complexes per APC, or high number of pMHCII^+^ APC, triggers persistent TCR signaling in thymocytes. Alternatively, highly expressed self-peptides may induce deletion because they are presented in the cortex to immature thymocytes that are more sensitive to deletion than mature thymocytes in the medulla (10, 11). Thus, according to current concepts, self-peptides with low or sparse presentation in the thymic medulla should form T_regitopes_.

A self-peptide derived from the α3 chain of type IV collagen (α3) forms a T_regitope_ when presented by the MHCII molecule, human leucocyte antigen (HLA)-DR1, but not when presented by HLA-DR15 (4). HLA genotype would not be expected to affect α3 expression, which has been observed in the thymic medulla in a pattern suitable for T_regitope_ formation (12). Although HLA-DR1 and HLA-DR15 both present the α3 self-peptide to T cells, the peptide anchor residues are offset by one position so that the TCR “sees” different amino acids of the peptide when it is presented by HLA-DR1 *versus* HLA-DR15 (4). The distinct fates of α3-specific CD4^+^ T cells are of special interest because humans and mice expressing HLA-DR15 are susceptible to Goodpasture’s disease, also known as anti-glomerular basement membrane disease, characterized by pro-inflammatory T cell responses towards α3/DR15 (13, 14). However, co-expression of HLA-DR1 induces development of α3/DR1-specific T-reg cells and prevents Goodpasture’s disease in a manner that depends on T-reg cells (4).

To account for the distinct fates of CD4^+^ T cells specific for α3/DR1 *versus* α3/DR15, and other findings, here we propose an extension to current concepts of thymic T-reg cell development. We postulate the potential of medullary pMHCII self-antigens to form a T_regitope_ can be extinguished when a high percentage of cognate antigen-specific thymocytes are deleted by encountering the same or similar pMHCII self-antigens in the cortex. We suggest antigen-specific CD4^+^ CD8^–^ (CD4 single-positive, CD4SP) thymocytes deliver signals that induce medullary thymic epithelial cells (mTEC) to “lock in” expression of their cognate self-antigen. Self-reactive CD4SP thymocytes may thereby generate a medullary niche for subsequent development of T-reg cells specific for the same self-antigen. Thus, antigen-specific T-reg niche size may be inversely related to the extent of cortical deletion of antigen-specific thymocyte populations. Implications of this extended model for the pathogenesis of organ-specific autoimmune diseases are discussed.

## Impact of Thymocyte Deletion in the Cortex on T-reg Selection in the Medulla

Thymocyte deletion has been dissected based on the maturation stage and/or the intrathymic location of the thymocytes undergoing deletion (15). Most CD4^+^ CD8^+^ (double positive, DP) thymocytes are located in the cortex, whereas CD4SP thymocytes migrate between cortex and medulla, preferentially residing in the medulla (16, 17). In models in which deletion occurs at the DP stage, increased numbers of apoptotic cells are found in the cortex (18–20), whereas deletion at the CD4SP stage results in increased numbers of apoptotic cells in the medulla (18). Thus, it is plausible that deletion of DP thymocytes occurs in the cortex and deletion of CD4SP thymocytes occurs in the medulla. However, DP CD69^+^ thymocytes can enter the medulla in a CCR4-dependent mechanism (21). In mixed chimeras, *Ccr4^–/–^* thymocytes are overrepresented in all TCR-signalled thymocyte subsets starting at the DP CD69^+^ stage (21). Those findings indicate CCR4 is required for normal deletion and suggest this deletion may occur in DP thymocytes inside the medulla. Still, considering the high frequency of thymocytes that undergo deletion at the DP stage (22–24), the relatively mild effect of CCR4 deficiency on deletion (21, 25) suggests that a substantial amount of deletion at the DP stage is independent of CCR4. While the relative contributions of the cortex and medulla to thymocyte deletion at the DP stage remain unclear, for conceptual clarity, in this Hypothesis and Theory article we have assumed that deletion at the DP stage occurs predominantly in the cortex, and we refer to this process as cortical deletion. Cortical deletion is widely considered to be inconsequential to T-reg selection because a thymocyte deleted in the cortex cannot directly affect events taking place in the medulla. However, we postulate that cortical deletion can affect T-reg selection by creating variation in the number of antigen-specific CD4SP thymocytes in the medulla.

An antigen-specific CD4^+^ T cell is typically identified by the binding of its TCR to a given pMHCII tetramer (26). In a naïve C57BL/6 (B6) mouse, the number of self-antigen-specific CD4SP thymocytes varies by 100-fold depending on the peptide embedded in the MHCII tetramer (27). Most of this effect arises from variation in the proportion of antigen-specific thymocytes that undergo deletion (28). Deletion of antigen-specific thymocytes need not be triggered by the antigen itself. For example, the IgM:I-A^b^-specific CD4SP thymocyte population in B6 mice is small (28). This population is ~ 8 times larger in mice that lack B cells–the only source of the IgM self-peptide–indicating IgM:I-A^b^ itself is required for the deletion of some IgM:I-A^b^-specific thymocytes. However, the IgM:I-A^b^-specific CD4SP thymocyte population is 450 times larger in mice with truly defective deletion due to MHCII expression being confined to cortical thymic epithelial cells, demonstrating that most IgM:I-A^b^-specific thymocytes can be deleted by self-antigens other than IgM:I-A^b^ (28). Enumerating CD4^+^ T cells specific for a panel of foreign pMHCII antigens revealed the extent of such “deletion *via* TCR cross-reactivity” correlates with the number of self-peptides with the same or similar TCR-exposed amino acids (29). Deletion of thymocytes expressing a cross-reactive TCR (30) is indistinguishable from deletion of thymocytes specific for ubiquitous self-antigen (20). Both are initiated at the DP stage and the thymocytes never reach the CD4SP stage at which Foxp3 is upregulated in developing T-reg cells (31, 32). Cortical deletion prevents cross-reactive and ubiquitously self-reactive thymocytes from developing into T-reg cells.

**Figure 1:**
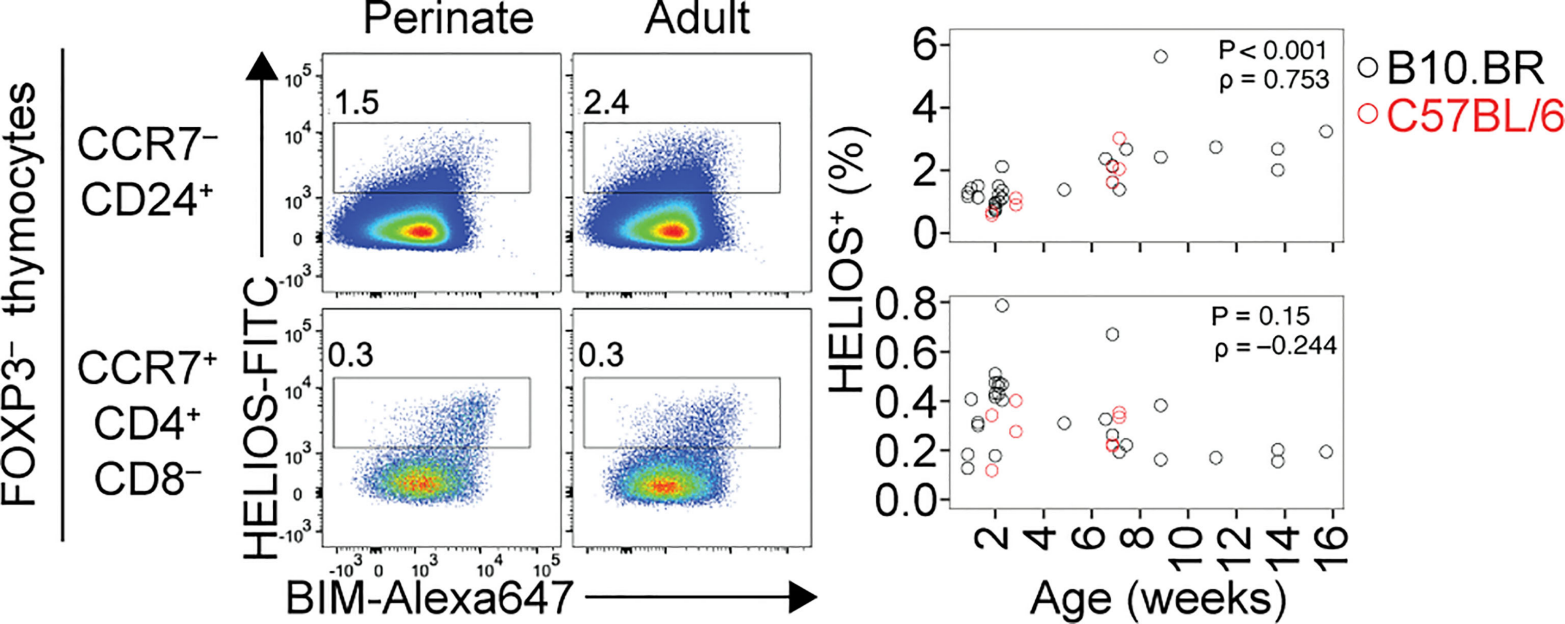
Age-dependent shift in cortical and medullary tolerance induction in the thymus. Flow cytometry plots (left) show HELIOS/BIM phenotypes of Foxp3^–^ thymocytes, divided into CCR7^–^ CD24^+^ (cortical) and CCR7^+^ CD4^+^ CD8^–^ (medullary) populations. Note that HELIOS and BIM tend to be co-expressed. Within each population, numbers on the plots show the percentage of HELIOS^+^ cells among all thymocytes, with graphs (right) showing results for multiple mice of the indicated ages and strains. Cortical tolerance appears less prominent during perinatal life, potentially enabling a higher frequency of strongly self-reactive thymocytes to develop into T-reg cells in the medulla in perinates compared to adults. P and rho values were determined using Spearman’s test for correlation.

The perinatal period is a critical time for immune tolerance (33). Perinatal T-reg cells are more effective than adult T-reg cells at preventing autoimmune disease provoked by Aire deficiency (34). The perinatal T-reg TCR repertoire is distinct from, and more diverse than, the adult T-reg TCR repertoire (34). The age-dependent change in T-reg selection is partly attributable to mTEC directly presenting a higher number of self-antigens in perinates than in adults (34). However, several findings suggest the extent of cortical deletion is also different in perinates and adults. The percentage of strongly TCR-signalled (Helios^+^) cells in the immature (CCR7^–^ CD24^+^) thymocyte population increases with age (**Figure 1**). This effect, which was reported previously (35), suggests cortical deletion is smaller in magnitude during the perinatal period than in adult life. This may be due to MHCII^high^ CD8α^+^ DC being less frequent in the perinatal thymus than in the adult thymus (34). Although cortical thymic epithelial cells can induce strong TCR signaling in some thymocytes (20, 36, 37), BM-derived APC (BM-APC), including DC, are present in the cortex (38, 39) and are required for normal cortical deletion (20, 36, 37, 40). An age-dependent change in the fate of thymocytes specific for one natural self-antigen has been documented (6). In mice with transgenic expression of the TCRβ chain from the Yae62 TCR (Yae62β-tg) (41), peptidyl arginine deiminase type IV (Padi4):I-A^b^ is a T_regitope_ at 1-3 weeks after birth; however, Padi4:I-A^b^-specific thymocytes are deleted at the DP stage or at the DP-CD4SP transition from 4 weeks after birth onwards (6). Analysis of *Padi4^–/–^* Yae62β-tg mice confirmed Padi4:I-A^b^-specific thymocytes undergo T-reg development in perinates and deletion in adults in response to Padi4 itself, with Padi4 expression in BM-APC sufficient to induce deletion in adult mice (6). Thymocytes with the potential to develop into T-reg cells can be deleted instead of developing into T-reg cells if they encounter a related self-peptide, or the cognate self-peptide itself, at the DP stage in the thymic cortex.

Events that occur within the DP stage in thymocytes that become T-reg cells have been puzzling to resolve. Commitment to the T-reg lineage within the DP stage was thought to be common, based on flow cytometry data indicating ~ 33% of Foxp3^+^ thymocytes in adult wild-type mice were DP cells (42). However, another study reported <10% of Foxp3^+^ thymocytes were DP cells including during the perinatal period (31). After rigorous exclusion of doublet events during flow cytometric analysis, <5% of Foxp3^+^ thymocytes had a DP phenotype (32). Accordingly, analysis of thymocytes that had incorporated a DNA label at the DP stage showed that Foxp3 upregulation predominantly occurs 4-8 days after label uptake, by which time the labelled cells have acquired a CCR7^+^ CD4SP phenotype (43) and moved to the medulla (44). Although we cannot exclude the possibility that commitment to the T-reg lineage can occur at the DP stage in the cortex, we favour the view that this usually occurs at the CCR7^+^ CD4SP stage when the thymocytes are in the medulla.

## Current Concepts of Developmental Niches for T-reg Cells in the Thymic Medulla

T-reg cells that develop in the thymus are thought to encounter their cognate self-antigen for the first time in the medulla (45, 46). This is plausible because the intra-thymic expression of some self-antigens, including tissue-restricted antigens (TRA), is confined to mTEC (47). Two major mTEC subsets are distinguished by expression of CCL21 (48), a chemokine that attracts CCR7^+^ thymocytes to the medulla (49), or the nuclear protein, Aire, which is required for normal expression of thousands of TRA by mTEC (50–52). Another nuclear protein, Fezf2, which is required for a distinct program of TRA expression independent of Aire (53), is expressed by CCL21^+^ mTEC and Aire^+^ mTEC (54, 55). Self-antigens expressed by mTEC can be taken up and presented by BM-APC (56, 57) and the presentation of some self-antigens to thymocytes is completely dependent on this mechanism (58, 59). While the mTEC population collectively expresses almost all protein-coding genes, the expression of individual genes varies widely both at the level of transcript abundance and in the frequency of mTECs that express the transcript (52). Many self-antigens are thought to be presented to thymocytes in small and discrete foci, which form a “mosaic” of developmental niches for antigen-specific T-reg cells in the medulla (45, 60).

This “mosaic” of self-antigen expression is shaped by proliferation, differentiation, and maturation of mTEC. Proliferating mTEC, which express many chromatin-modifying factors and some TRA, give rise to cells that express Aire and a higher number of TRAs per mTEC (54, 61). Whether CCL21^+^ mTEC are precursors or progeny of proliferating mTEC remains unclear (35, 54, 61). The current concept is that an individual mTEC expresses different sets of self-antigens over its lifetime (61–63). In support of this “colinear differentiation” model (62), an individual mTEC can switch off expression of one self-antigen and switch on expression of another (62, 64). In addition, single-cell RNA sequencing identified sets of self-antigens that were co-expressed in multiple mTEC (61, 63, 65). In this model, the presence of cells spanning all mTEC subsets and all maturation stages is necessary and sufficient for the expression of a full “mosaic” of self-antigens in the medulla.

The thymic medulla is smaller in mice lacking CD4SP thymocytes compared to wild-type mice or mice lacking CD8SP thymocytes (66). Development of the mature mTEC population, defined by high expression of MHCII and the costimulatory molecule CD80, and comprising an Aire^+^ subset, requires cognate interactions between the TCR on CD4SP thymocytes and pMHCII on mTECs (67). CD4SP thymocytes express the ligands for RANK, CD40, and LTβR, which are cell-surface receptors necessary for mTEC maturation (66, 68, 69). Anti-RANK ligand (RANKL) antibody treatment and the absence of self-reactive CD4SP thymocytes both cause deficiency of Aire^+^ mTEC, whereas CCL21^+^ mTEC remain largely intact (54, 55). Notably, anti-RANKL antibodies diminish the frequency of proliferating cells in the mTEC population (54, 70), whereas the absence of self-reactive CD4SP thymocytes does not (55). It is possible that invariant NKT cells provide enough RANKL to support mTEC proliferation (71). However, normal transition from the proliferating stage to the Aire^+^ stage in mTEC development requires signals uniquely provided during cognate interactions with self-reactive CD4SP thymocytes (66). Similarly, normal development of mature thymic DCs requires cognate TCR-pMHCII interactions with CD4SP thymocytes (72).

CD4SP thymocytes also contribute to thymic T-reg cell niches by producing IL-2 (73, 74). Consumption of this IL-2 prevents deletion of strongly TCR-signalled CD4SP thymocytes (75) and enables these T-reg precursors to upregulate Foxp3 expression (76, 77). CD4SP thymocytes are thus both inducers and “clients” of antigen-specific T-reg cell niches in the thymic medulla.

## A Role for CD4SP Thymocytes in Generating the T-reg Niche Containing Their Cognate Self-Antigen?

To this picture we wish to add the hypothesis that strongly self-reactive CD4SP thymocytes foment the niche containing their cognate self-antigen. Sustained, repetitive, cognate interactions between CD4SP thymocytes and mTEC may be necessary for the survival of post-cycling Aire^+^ mTEC and may induce the mTEC to pause or arrest its “colinear differentiation” program. In other words, these interactions may “lock in” continued expression of those self-antigens that the mTEC is expressing at the time. Currently available data do not exclude this extended model. During the post-cycling Aire^+^ stage, different studies found the mean number of TRAs expressed per Aire^+^ mTEC remained constant (61) or increased by a factor of only two (54). Multiple mTEC that co-express sets of self-antigens may be “daughters of the same epithelial cell progenitor” (65).

**Figure 2:**
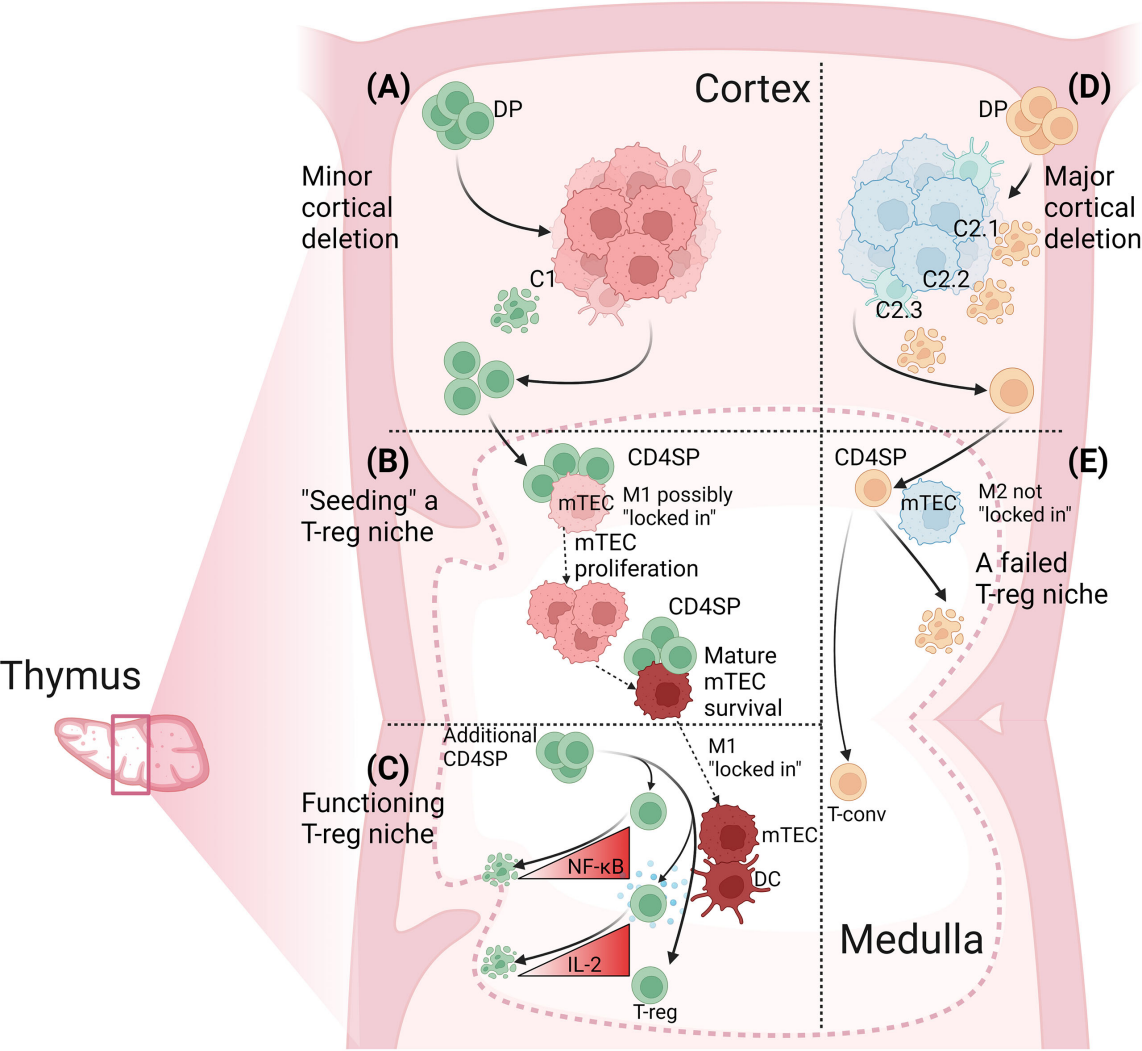
Determinants of antigen-specific T-reg niche size in the thymus. **(A)** The extent of cortical deletion is minor when the relatedness between cortical and medullary peptides is low. Out of four DP thymocytes specific for a medullary peptide, M1, one is deleted by a related cortical peptide, C1, and the other three progress to the CD4SP stage and migrate into the medulla. **(B)** Cognate CD4SP-mTEC interactions “seed” the T-reg niche. CD4SP thymocytes could mediate this effect by interacting with immature mTEC prior to the onset of proliferation and/or by promoting mTEC survival at the post-cycling stage through sustained engagement of RANK, CD40 and LTβR expressed on mTEC. The key outcome is that M1-specific CD4SP thymocytes induce the M1-presenting mTEC to “lock in” M1 expression. **(C)** A functioning T-reg niche. By now, the mature M1-expressing mTEC has high expression of MHCII, CD80, Aire and the protein from which the M1 peptide is derived. M1-specific CD4SP thymocytes also induce local DC to increase MHCII and CD80 expression. M1-specific CD4SP thymocytes also produce IL-2. However, most M1-specific CD4SP thymocytes undergo deletion due to insufficient NF-κB activation or insufficient IL-2 consumption. Rare M1-specific CD4SP thymocytes activate sufficient NF-κB and consume sufficient IL-2 to survive, upregulate Foxp3 and progress to the next stage of T-reg development. **(D)** The extent of cortical deletion is major when the relatedness between cortical and medullary peptides is high. Out of four DP thymocytes specific for medullary peptide, M2, three are deleted in the cortex by related self-peptides, C2.1, C2.2 and C2.3, and only one progresses to the CD4SP stage and migrates into the medulla. **(E)** A failed T-reg niche. The number of M2-specific CD4SP thymocytes is too low to provide inductive signals to the M2-expressing mTEC. The mTEC may switch off expression of M2 and switch on expression of different self-antigens. Activation of M2-specific CD4SP thymocytes is insufficient to induce T-reg development but may be sufficient to induce deletion of some cells. No M2-specific T-reg niche forms and the small M2-specific CD4SP population develops into T-conv cells. Figure created with BioRender.com.

Through this process, antigen-specific CD4SP thymocytes may seed a niche for the development of T-reg specific for the same self-antigen or another self-antigen in the same co-expression “module” (61). Generating a functional T-reg niche requires collaboration between CD4SP thymocytes because no single cell can fulfil all functions required of CD4SP thymocytes. These functions include: (i) to induce the post-cycling Aire^+^ mTEC to survive and “lock in” its current self-antigen expression profile; (ii) to induce the mTEC and local DC to upregulate antigen-presenting and costimulatory molecules; (iii) to produce IL-2; and (iv) to develop from a naïve CD4SP thymocyte into a T-reg precursor and then into a T-reg cell. At another level, there is inter-niche competition because the medullary volume limits the number of niches present at a given time. Success in this inter-niche competition may be proportional to the extent of intra-niche collaboration, which is in turn dictated by the size of the antigen-specific CD4SP thymocyte population in the medulla (**Figure 2**).

This extended hypothesis can accommodate some unexplained findings. Two TCRs, called DO11 and N7, can facilitate T-reg development in mice expressing the neo-self-antigen, ovalbumin (OVA) (78). For these two TCRs, as had been observed in other models (2, 79), antigen-specific CD4SP thymocyte population size and Foxp3^+^ cell frequency were inversely related, consistent with T-reg development being constrained by OVA:I-A^d^ availability (78). Surprisingly, and in contrast to the inverse relationship, T-reg development failed when the CD4SP thymocyte populations expressing the DO11 or N7 TCR were very small (78). We suggest the OVA:I-A^d^-specific population size had a lower limit, below which intra-clonal collaboration between the TCR-transgenic CD4SP thymocytes was insufficient to generate a niche for effective OVA:I-A^d^-specific T-reg development.

Our hypothesis also accommodates complementary findings based on CD4^+^ T cell responses to natural self-antigens. Although T-reg cell populations specific for myelin oligodendrocyte glycoprotein (MOG):I-A^b^ can be expanded in B6 mice immunized with MOG peptide (8, 80), MOG:I-A^b^-specific T-reg cells are rare in naïve B6 mice (27, 81). A notably different phenotype is observed in B6.*Kaa* mice (81), which express a transgenic TCRβ chain repetitively found in MOG:I-A^b^-specific T cells (82). Compared to naïve B6 mice, naïve B6.*Kaa* mice have 4 times more MOG:I-A^b^-specific CD4^+^ T-conv cells and 16 times more MOG:I-A^b^-specific CD4^+^ T-reg cells (81). Furthermore, proliferation and suppressive function of MOG:I-A^b^-specific CD4^+^ T-reg cells were demonstrable in T-reg populations from naïve B6.*Kaa* mice but not from naïve B6 mice (81). Similar findings were made in an analogous study of CD4^+^ T cells specific for proteolipid protein (PLP):I-A^b^ (83). In the latter study, comparison of *Plp1* ^+/+^ and *Plp1* ^–/–^ mice on the TCRβ-transgenic background provided the additional insight that PLP expression induced the post-thymic expansion of PLP:I-A^b^-specific CD4^+^ T-reg and Foxp3^–^ FR4^+^ CD73^+^ anergic (84) populations, but not the Foxp3^–^ FR4^–^ CD73^–^ naïve T-conv cell population (83). In both studies (81, 83), enlarging the antigen-specific CD4SP thymocyte population *via* a TCRβ transgene expanded the antigen-specific T-reg cell niche.

**Figure 3:**
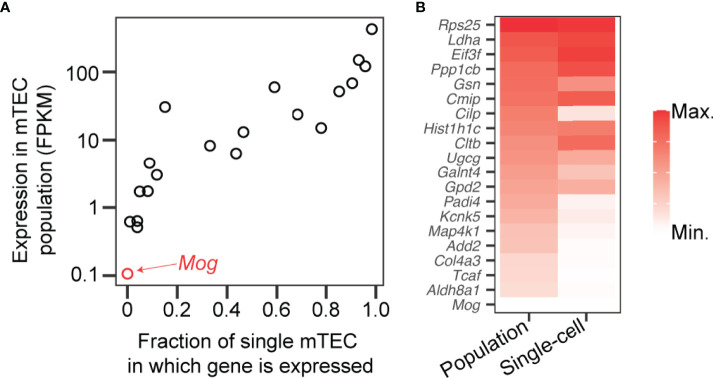
Low expression of Mog compared to self-antigens known to induce T-reg cell development in the thymus. **(A)** Transcripts encoding myelin oligodendrocyte glycoprotein (*Mog*) and 19 self-antigens known to induce thymic T-reg development (4–6) were measured by RNA sequencing of mTEC at the population level (*y*-axis) (52) and in 305 single cells (*x*-axis) (63). FPKM, fragments per kilobase of exon per million mapped fragments. **(B)** Transcription levels of the 20 genes represented in **(A)** in the mTEC population (left) and in single mTEC (right) with red shading according to the scales shown on the axes in **(A)**.

A challenge to our hypothesis is that the MOG:I-A^b^-specific CD4^+^ population in naïve B6 mice is relatively large, close to the top of the spectrum of foreign pMHCII-specific population sizes (29). Our hypothesis would predict the large MOG:I-A^b^-specific CD4SP thymocyte population in B6 mice ought to establish a MOG:I-A^b^-specific T-reg cell niche. To compare the intra-thymic expression of MOG with natural self-antigens known to form T_regitopes_ (4–6), we analyzed data from two studies that conducted RNA sequencing on mTEC samples (52, 63). In this panel of 20 self-antigens, both studies found MOG had the lowest expression in mTECs (**Figure 3**), suggesting low “basal” MOG expression in the thymic medulla might limit MOG:I-A^b^-specific T-reg niche generation in B6 mice. An initial test of our hypothesis would be to compare the abundance of MOG transcripts in mTEC from B6 *versus* B6.*Kaa* mice. We predict the unusually large MOG:I-A^b^-specific CD4SP thymocyte population in B6.*Kaa* mice would result in higher MOG transcription in the mTEC population. Our hypothesis would also predict the presence of B6.*Kaa* thymocytes should enhance T-reg development in co-resident wild-type thymocytes in mixed chimeras.

## Impact of Thymocyte Deletion in the Medulla on T-reg Selection

Outside the thymus, in a self-tolerant and functional immune system, T-reg cells are thought to outcompete T-conv cells for APCs that are presenting self-antigens, whereas the reverse would apply for APCs presenting foreign antigens (85). Theoretically, self-tolerance should be most robust if the thymus selected the most self-reactive thymocytes into the T-reg lineage, in order to maximize the difference in self-reactivity between T-reg and T-conv cells. However, this is not observed experimentally. Antigen-specific T-reg cells bind fewer pMHCII tetramer molecules per cell than some antigen-specific T-conv cells in mice lacking the self-antigen (8, 80). This suggests the self-antigen deletes those thymocytes that express the most self-reactive TCRs. Consistent with this conclusion, the half-lives and functional avidities of 10 Padi4:I-A^b^-specific TCRs indicated the high, intermediate and low ranges of TCR self-reactivity induced deletion, (perinatal) T-reg cell development, and T-conv cell development, respectively (6). However, there is also compelling evidence that CD4SP thymocytes can undergo deletion as a result of their TCR self-reactivity being too low for T-reg development (86). Accordingly, in a panel of 4 PLP:I-A^b^-specific TCRs, the TCRs with the highest and lowest functional avidity induced deletion, whereas the 2 TCRs with intermediate functional avidity induced T-reg development (83). The TCR self-reactivity most conducive to T-reg development would appear to be “sandwiched” between two ranges of TCR self-reactivity that induce deletion in the thymic medulla.

After CD4SP thymocytes initiate strong TCR signaling in the medulla, the thymocyte-intrinsic pathways required to prevent deletion change as the thymocyte matures. Canonical NF-κB activation prevents deletion within the Foxp3^–^ T-reg precursor stage (22, 87–89), whereas IL-2 signaling prevents deletion at a later stage, close to the time of Foxp3 upregulation (75). DOCK8 inhibits deletion at both of these stages (90). For CD4SP thymocytes inside a medullary T-reg cell niche, survival requires signaling that is not required for deletion. Evidence that most of these cells are deleted (22, 75) suggests those cells that complete T-reg development are rigorously selected.

CD4SP thymocytes can also develop into T-reg cells *via* a developmental pathway that includes a Foxp3^+^ CD25^–^ T-reg precursor stage (31, 91). Compared to Foxp3^–^ CD25^+^ T-reg precursors, Foxp3^+^ CD25^–^ T-reg precursors take longer to develop, tend to have lower TCR self-reactivity and are less susceptible to deletion (92). This alternative pathway may be used by TCRs such as the OVA:I-A^d^-specific TCR called R4 (78) and another TCR called G113 (2). The R4 and G113 TCRs still induce T-reg development when they are expressed by very few CD4SP thymocytes (2, 78), implying intra-clonal collaboration is unnecessary for these TCRs to support T-reg development. Unlike the DO11 and N7 TCRs, the R4 and G113 TCRs do not induce measurable deletion (2, 78), suggesting only TCRs that trigger deletion require the antigen-specific CD4SP thymocyte population size to exceed a lower limit in order to induce T-reg development. CD4SP thymocytes with a TCR self-reactivity too low to induce deletion would be expected to have a longer lifespan in the thymic medulla, which may increase their probability of finding a pre-existing, functional T-reg niche, and surviving long enough to upregulate Foxp3. Whether an antigen-specific CD4SP Foxp3^–^ thymocyte is at risk of deletion or not, it can still contribute to the antigen-specific T-reg cell niche by providing inductive signals to APCs and by producing IL-2 (**Figure 2**).

The adult thymus also contains recirculating or thymus-resident T-reg cells (93), which may impact *de novo* thymic T-reg development. GK-transgenic mice, which have few peripheral CD4^+^ T cells and few non-nascent T-reg cells in the thymus due to transgenic expression of an anti-CD4 antibody, have a slightly higher frequency of Foxp3^+^ thymocytes than wild-type mice (94). Non-nascent T-reg cells may thus limit *de novo* thymic T-reg development by competing for limiting IL-2 (94, 95). However, *de novo* thymic T-reg development is not reduced in mice with enlarged non-nascent thymic T-reg cell populations (96, 97). As non-nascent T-reg cells express more *Tnfsf11* and *Cd40lg* transcripts (which encode RANKL and CD40L, respectively) than nascent thymic T-reg cells (94), they may also positively affect the thymic T-reg niche by providing inductive signals to APC.

## Discussion

Certain self-antigens reproducibly “select” CD4SP thymocytes to enter the T-reg lineage (4–6). We refer to these self-antigens as T_regitopes_. Here, we postulate a mechanism that operates in the opposite direction, wherein CD4SP thymocytes “select” self-antigens to become T_regitopes_. This hypothesis draws on evidence that the major T-reg-inducing APC subsets in the thymus, mTEC and DC, require cognate TCR-pMHCII-dependent interactions with CD4SP thymocytes in order to form mature populations (55, 66, 67, 72). We propose CD4SP thymocytes deliver signals that promote mature mTEC survival and “lock in” the set of self-antigens being expressed by the mTEC at the time. This endows a self-reactive CD4SP thymocyte with the ability to generate a medullary niche containing its cognate self-antigen, enabling subsequent development of T-reg cells specific for the same self-antigen. We propose the “mosaic” of antigen-specific T-reg niches in the thymic medulla (45, 60) is not predetermined but is shaped by the antigen specificities of CD4SP thymocytes in the medulla. Deletion creates variation in the number of CD4SP thymocytes specific for different self-antigens (27, 28). The size of the antigen-specific T-reg niche in the medulla may be inversely related to the extent of cortical deletion of antigen-specific thymocyte populations (**Figure 2**).

It is unclear why α3/DR1 is a T_regitope_, whereas α3/DR15 is not (4). Although the thymus was not analyzed, peripheral CD4^+^ T cell populations in mice expressing these human HLA molecules contained a higher frequency of α3/DR1-specific cells than α3/DR15-specific cells (4). This difference may be due to greater cortical deletion of α3/DR15-specific thymocytes compared to α3/DR1-specific cells. If so, then this cortical deletion is unlikely to be triggered by α3/DR15 itself, as the α3 protein is sparsely expressed in the thymic medulla (12). Furthermore, α3/DR1 is a T_regitope_, suggesting the α3 self-peptide is not displayed to cortical thymocytes. We infer that cortical deletion of α3/DR15-specific thymocytes is mediated by related self-peptides with similar TCR-exposed residues (29). An initial test of this hypothesis may involve enumerating antigen-specific thymocytes at distinct maturation stages, as described (6). Our hypothesis would predict the presence of DR1 ought to “lock in” α3 expression and augment selection of α3/DR15-specific T-reg cells. However, DR1 expression did not affect the α3/DR15-specific T-reg or T-conv cell frequency in DR1^+^ DR15^+^ mice compared to DR15^+^ mice (4). Differential affinity of the α3 peptide for DR1 *versus* DR15 may lead to differences in the quantity of the two pMHCII complexes. Alternatively, differences in the chemistry of the different TCR-exposed peptide residues may lead to differences in the TCR affinity distribution of CD4SP thymocytes specific for the two pMHCII complexes. These differences may bias T-reg development towards the α3/DR1 T_regitope_ despite co-expression of the two MHC alleles.

Associations between human autoimmune diseases and particular MHC alleles (98) indicate a role for TCR-peptide-MHC interactions in pathogenesis. The current paradigm is that autoimmune diseases are mediated by pro-inflammatory T-conv cells specific for self-peptides presented by disease-associated MHC alleles (99). Interestingly, compared to TCR-peptide-MHC interactions elicited by infection or immunization, some autoimmune interactions have unusual features, including atypical positioning of the TCR or the self-peptide, post-translational self-peptide modifications and self-peptide fusions (99). These findings shed light on the nature of inappropriate self-antigen recognition by T cells. However, they do not explain why only some people with disease-associated MHC alleles develop autoimmune disease. This implies the action of an additional predisposing factor, such as the absence of an antigen-specific T-reg cell population that would otherwise prevent autoimmune disease. The association between autoimmune diseases and particular MHC alleles may reflect the lack of an organ-specific self-peptide that can form a T_regitope_ when presented by the disease-associated MHCII allele. We refer to this as T_regitope_ deficiency.

Other genetic factors may combine with a disease-associated MHCII allele to avert or contribute to T_regitope_ deficiency. Co-expression of an MHCII allele that can form a T_regitope_ can avert T_regitope_ deficiency, as exemplified with HLA-DR1 in Goodpasture’s disease (4, 13). T_regitope_ sufficiency or deficiency may explain why pairs of HLA haplotypes are associated with a decreased or increased risk of autoimmune diseases beyond the additive contributions of each haplotype (100). In addition, T_regitope_ deficiency may require a high relatedness between (at least) two self-peptides, one presented in the cortex and the other in the medulla, a situation that may extinguish the potential of the medullary self-peptide to serve as a T_regitope_. Hence, T_regitope_ deficiency would be expected in only a subset of individuals who inherit a disease-associated MHCII allele, providing an explanation for why most such individuals never develop autoimmune disease.

## Data Availability Statement

The raw data supporting the conclusions of this article will be made available by the authors, without undue reservation.

## Ethics Statement

The animal study was reviewed and approved by The Animal Experimentation Ethics Committee of the Australian National University.

## Author Contributions

CW and SD jointly prepared figures and wrote the paper. All authors contributed to the article and approved the submitted version.

## Funding

CW and SD received support from the Queensland University of Technology. SD received support from the National Health and Medical Research Council (grant 1188589).

## Conflict of Interest

The authors declare that the research was conducted in the absence of any commercial or financial relationships that could be construed as a potential conflict of interest.

## Publisher’s Note

All claims expressed in this article are solely those of the authors and do not necessarily represent those of their affiliated organizations, or those of the publisher, the editors and the reviewers. Any product that may be evaluated in this article, or claim that may be made by its manufacturer, is not guaranteed or endorsed by the publisher.

## References

[1] Sakaguchi S Mikami N Wing JB Tanaka A Ichiyama K Ohkura N. Regulatory T Cells and Human Disease. Annu Rev Immunol (2020) 38:541. doi: 10.1146/annurev-immunol-042718-041717 32017635

[2] Bautista JL Lio CW Lathrop SK Forbush K Liang Y Luo J. Intraclonal Competition Limits the Fate Determination of Regulatory T Cells in the Thymus. Nat Immunol (2009) 10:610–17. doi: 10.1038/ni.1739 PMC2756247 19430476

[3] Levine AG Arvey A Jin W Rudensky AY. Continuous Requirement for the TCR in Regulatory T Cell Function. Nat Immunol (2014) 15:1070–78. doi: 10.1038/ni.3004 PMC4205268 25263123

[4] Ooi JD Petersen J Tan YH Huynh M Willett ZJ Ramarathinam SH. Dominant Protection From HLA-Linked Autoimmunity by Antigen-Specific Regulatory T Cells. Nature (2017) 545:243–47. doi: 10.1038/nature22329 PMC5903850 28467828

[5] Leonard JD Gilmore DC Dileepan T Nawrocka WI Chao JL Schoenbach MH. Identification of Natural Regulatory T Cell Epitopes Reveals Convergence on a Dominant Autoantigen. Immunity (2017) 47:107–17. doi: 10.1016/j.immuni.2017.06.015 PMC5562039 28709804

[6] Stadinski BD Blevins SJ Spidale NA Duke BR Huseby PG Stern LJ. A Temporal Thymic Selection Switch and Ligand Binding Kinetics Constrain Neonatal Foxp3^+^ T_reg_ Cell Development. Nat Immunol (2019) 20:1046–58. doi: 10.1038/s41590-019-0414-1 PMC7039339 31209405

[7] Ferrante A. For Many But Not for All: How the Conformational Flexibility of the Peptide/MHCII Complex Shapes Epitope Selection. Immunol Res (2013) 56:85–95. doi: 10.1007/s12026-012-8342-2 22753017 PMC4197051

[8] Malhotra D Linehan JL Dileepan T Lee YJ Purtha WE Lu JV. Tolerance is Established in Polyclonal CD4^+^ T Cells by Distinct Mechanisms, According to Self-Peptide Expression Patterns. Nat Immunol (2016) 17:187–95. doi: 10.1038/ni.3327 PMC4718891 26726812

[9] Legoux FP Lim JB Cauley AW Dikiy S Ertelt J Mariani TJ. CD4^+^ T Cell Tolerance to Tissue-Restricted Self Antigens Is Mediated by Antigen-Specific Regulatory T Cells Rather Than Deletion. Immunity (2015) 43:896–08. doi: 10.1016/j.immuni.2015.10.011 PMC4654997 26572061

[10] Ebert PJ Ehrlich LI Davis MM. Low Ligand Requirement for Deletion and Lack of Synapses in Positive Selection Enforce the Gauntlet of Thymic T Cell Maturation. Immunity (2008) 29:734–45. doi: 10.1016/j.immuni.2008.09.014 PMC3762485 18993085

[11] Peterson DA DiPaolo RJ Kanagawa O Unanue ER. Cutting Edge: Negative Selection of Immature Thymocytes by a Few Peptide-MHC Complexes: Differential Sensitivity of Immature and Mature T Cells. J Immunol (1999) 162:3117–20. 10092759

[12] Wong D Phelps RG Turner AN. The Goodpasture Antigen is Expressed in the Human Thymus. Kidney Int (2001) 60:1777–83. doi: 10.1046/j.1523-1755.2001.00014.x 11703595

[13] Phelps RG Rees AJ. The HLA Complex in Goodpasture's Disease: A Model for Analyzing Susceptibility to Autoimmunity. Kidney Int (1999) 56:1638–53. doi: 10.1046/j.1523-1755.1999.00720.x 10571772

[14] Ooi JD Chang J O'Sullivan KM Pedchenko V Hudson BG Vandenbark AA. The HLA-DRB1*15:01-Restricted Goodpasture's T Cell Epitope Induces GN. J Am Soc Nephrol (2013) 24:419–31. doi: 10.1681/ASN.2012070705 PMC3582203 23411782

[15] Daley SR Teh C Hu DY Strasser A Gray DHD. Cell Death and Thymic Tolerance. Immunol Rev (2017) 277:9–20. doi: 10.1111/imr.12532 28462532

[16] Ehrlich LI Oh DY Weissman IL Lewis RS. Differential Contribution of Chemotaxis and Substrate Restriction to Segregation of Immature and Mature Thymocytes. Immunity (2009) 31:986–98. doi: 10.1016/j.immuni.2009.09.020 PMC4106268 19962328

[17] Halkias J Melichar HJ Taylor KT Ross JO Yen B Cooper SB. Opposing Chemokine Gradients Control Human Thymocyte Migration in Situ. J Clin Invest (2013) 123:2131–42. doi: 10.1172/JCI67175 PMC3635739 23585474

[18] Douek DC Corley KT Zal T Mellor A Dyson PJ Altmann DM. Negative Selection by Endogenous Antigen and Superantigen Occurs at Multiple Thymic Sites. Int Immunol (1996) 8:1413–20. doi: 10.1093/intimm/8.9.1413 8921419

[19] Liblau RS Tisch R Shokat K Yang X Dumont N Goodnow CC. Intravenous Injection of Soluble Antigen Induces Thymic and Peripheral T-Cells Apoptosis. Proc Natl Acad Sci USA (1996) 93:3031–36. doi: 10.1073/pnas.93.7.3031 PMC39756 8610163

[20] McCaughtry TM Baldwin TA Wilken MS Hogquist KA. Clonal Deletion of Thymocytes can Occur in the Cortex With No Involvement of the Medulla. J Exp Med (2008) 205:2575–84. doi: 10.1084/jem.20080866 PMC2571932 18936237

[21] Hu Z Lancaster JN Sasiponganan C Ehrlich LI. CCR4 Promotes Medullary Entry and Thymocyte-Dendritic Cell Interactions Required for Central Tolerance. J Exp Med (2015) 212:1947–65. doi: 10.1084/jem.20150178 PMC4612092 26417005

[22] Daley SR Hu DY Goodnow CC. Helios Marks Strongly Autoreactive CD4^+^ T Cells in Two Major Waves of Thymic Deletion Distinguished by Induction of PD-1 or NF-κb. J Exp Med (2013) 210:269–85. doi: 10.1084/jem.20121458 PMC3570102 23337809

[23] Sinclair C Bains I Yates AJ Seddon B. Asymmetric Thymocyte Death Underlies the CD4:CD8 T-Cell Ratio in the Adaptive Immune System. Proc Natl Acad Sci USA (2013) 110:E2905–14. doi: 10.1073/pnas.1304859110 PMC3732981 23858460

[24] Stritesky GL Xing Y Erickson JR Kalekar LA Wang X Mueller DL. Murine Thymic Selection Quantified Using a Unique Method to Capture Deleted T Cells. Proc Natl Acad Sci U S A (2013) 110:4679–84. doi: 10.1073/pnas.1217532110 PMC3606987 23487759

[25] Cowan JE McCarthy NI Parnell SM White AJ Bacon A Serge A. Differential Requirement for CCR4 and CCR7 During the Development of Innate and Adaptive αβt Cells in the Adult Thymus. J Immunol (2014) 193:1204–12. doi: 10.4049/jimmunol.1400993 PMC4105241 24990081

[26] Moon JJ Chu HH Pepper M McSorley SJ Jameson SC Kedl RM. Naive CD4^+^ T Cell Frequency Varies for Different Epitopes and Predicts Repertoire Diversity and Response Magnitude. Immunity (2007) 27:203–13. doi: 10.1016/j.immuni.2007.07.007 PMC2200089 17707129

[27] Watanabe M Lu Y Breen M Hodes RJ. B7-CD28 Co-Stimulation Modulates Central Tolerance *via* Thymic Clonal Deletion and Treg Generation Through Distinct Mechanisms. Nat Commun (2020) 11:6264. doi: 10.1038/s41467-020-20070-x 33293517 PMC7722925

[28] Chu HH Moon JJ Kruse AC Pepper M Jenkins MK. Negative Selection and Peptide Chemistry Determine the Size of Naive Foreign Peptide-MHC Class II-Specific CD4^+^ T Cell Populations. J Immunol (2010) 185:4705–13. doi: 10.4049/jimmunol.1002276 PMC3510669 20861357

[29] Nelson RW Beisang D Tubo NJ Dileepan T Wiesner DL Nielsen K. T Cell Receptor Cross-Reactivity Between Similar Foreign and Self Peptides Influences Naive Cell Population Size and Autoimmunity. Immunity (2015) 42:95–107. doi: 10.1016/j.immuni.2014.12.022 25601203 PMC4355167

[30] McDonald BD Bunker JJ Erickson SA Oh-Hora M Bendelac A. Crossreactive αβ T Cell Receptors Are the Predominant Targets of Thymocyte Negative Selection. Immunity (2015) 43:859–69. doi: 10.1016/j.immuni.2015.09.009 PMC4654978 26522985

[31] Fontenot JD Dooley JL Farr AG Rudensky AY. Developmental Regulation of Foxp3 Expression During Ontogeny. J Exp Med (2005) 202:901–6. doi: 10.1084/jem.20050784 PMC2213175 16203863

[32] Lee HM Hsieh CS. Rare Development of Foxp3^+^ Thymocytes in the CD4^+^CD8^+^ Subset. J Immunol (2009) 183:2261–66. doi: 10.4049/jimmunol.0901304 PMC2912293 19620303

[33] Guerau-de-Arellano M Martinic M Benoist C Mathis D. Neonatal Tolerance Revisited: A Perinatal Window for Aire Control of Autoimmunity. J Exp Med (2009) 206:1245–52. doi: 10.1084/jem.20090300 PMC2715060 19487417

[34] Yang S Fujikado N Kolodin D Benoist C Mathis D. Immune Tolerance. Regulatory T Cells Generated Early in Life Play a Distinct Role in Maintaining Self-Tolerance. Science (2015) 348:589–94. doi: 10.1126/science.aaa7017 PMC4710357 25791085

[35] Baran-Gale J Morgan MD Maio S Dhalla F Calvo-Asensio I Deadman ME. Ageing Compromises Mouse Thymus Function and Remodels Epithelial Cell Differentiation. Elife (2020) 9. doi: 10.7554/eLife.56221 PMC7490013 32840480

[36] Wirasinha RC Chan A Yap JY Hu DY Teh CE Gray DHD. Deletion of Self-Reactive CCR7- Thymocytes in the Absence of MHC Expression on Thymic Epithelial Cells. Cell Death Differ (2019) 26:2727–39. doi: 10.1038/s41418-019-0331-8 PMC7224288 31019259

[37] Kurd NS Hoover A Yoon J Weist BM Lutes L Chan SW. Factors That Influence the Thymic Selection of CD8αα Intraepithelial Lymphocytes. Mucosal Immunol (2021) 14:68–79. doi: 10.1038/s41385-020-0295-5 32483197 PMC10443950

[38] Raviola E Karnovsky MJ. Evidence for a Blood-Thymus Barrier Using Electron-Opaque Tracers. J Exp Med (1972) 136:466–98. doi: 10.1084/jem.136.3.466 PMC2139259 4115129

[39] Ladi E Schwickert TA Chtanova T Chen Y Herzmark P Yin X. Thymocyte-Dendritic Cell Interactions Near Sources of CCR7 Ligands in the Thymic Cortex. J Immunol (2008) 181:7014–23. doi: 10.4049/jimmunol.181.10.7014 18981121

[40] Melichar HJ Ross JO Herzmark P Hogquist KA Robey EA. Distinct Temporal Patterns of T Cell Receptor Signaling During Positive Versus Negative Selection in Situ. Sci Signal (2013) 6::ra92. doi: 10.1126/scisignal.2004400 24129702 PMC4078262

[41] Stadinski BD Trenh P Smith RL Bautista B Huseby PG Li G. A Role for Differential Variable Gene Pairing in Creating T Cell Receptors Specific for Unique Major Histocompatibility Ligands. Immunity (2011) 35:694–704. doi: 10.1016/j.immuni.2011.10.012 22101158 PMC3253227

[42] Liston A Nutsch KM Farr AG Lund JM Rasmussen JP Koni PA. Differentiation of Regulatory Foxp3^+^ T Cells in the Thymic Cortex. Proc Natl Acad Sci USA (2008) 105:11903–08. doi: 10.1073/pnas.0801506105 PMC2575273 18695219

[43] Hu DY Yap JY Wirasinha RC Howard DR Goodnow CC Daley SR. A Timeline Demarcating Two Waves of Clonal Deletion and Foxp3 Upregulation During Thymocyte Development. Immunol Cell Biol (2016) 94:357–66. doi: 10.1038/icb.2015.95 26510893

[44] Penit C. *In Vivo* Thymocyte Maturation. BUdR Labeling of Cycling Thymocytes and Phenotypic Analysis of Their Progeny Support the Single Lineage Model. J Immunol (1986) 137:2115–21. 3093565

[45] Klein L Robey EA Hsieh CS. Central CD4^+^ T Cell Tolerance: Deletion Versus Regulatory T Cell Differentiation. Nat Rev Immunol (2019) 19:7–18. doi: 10.1038/s41577-018-0083-6 30420705

[46] Savage PA Klawon DEJ Miller CH. Regulatory T Cell Development. Annu Rev Immunol (2020) 38:421–53. doi: 10.1146/annurev-immunol-100219-020937 31990619

[47] Derbinski J Schulte A Kyewski B Klein L. Promiscuous Gene Expression in Medullary Thymic Epithelial Cells Mirrors the Peripheral Self. Nat Immunol (2001) 2:1032–39. doi: 10.1038/ni723 11600886

[48] Lkhagvasuren E Sakata M Ohigashi I Takahama Y. Lymphotoxin β Receptor Regulates the Development of CCL21-Expressing Subset of Postnatal Medullary Thymic Epithelial Cells. J Immunol (2013) 190:5110–17. doi: 10.4049/jimmunol.1203203 23585674

[49] Ueno T Saito F Gray DH Kuse S Hieshima K Nakano H. CCR7 Signals are Essential for Cortex-Medulla Migration of Developing Thymocytes. J Exp Med (2004) 200:493–05. doi: 10.1084/jem.20040643 PMC2211934 15302902

[50] Anderson MS Venanzi ES Klein L Chen Z Berzins SP Turley SJ. Projection of an Immunological Self Shadow Within the Thymus by the Aire Protein. Science (2002) 298:1395–01. doi: 10.1126/science.1075958 12376594

[51] Derbinski J Gabler J Brors B Tierling S Jonnakuty S Hergenhahn M. Promiscuous Gene Expression in Thymic Epithelial Cells is Regulated at Multiple Levels. J Exp Med (2005) 202:33–45. doi: 10.1084/jem.20050471 15983066 PMC2212909

[52] Sansom SN Shikama-Dorn N Zhanybekova S Nusspaumer G Macaulay IC Deadman ME. Population and Single-Cell Genomics Reveal the Aire Dependency, Relief From Polycomb Silencing, and Distribution of Self-Antigen Expression in Thymic Epithelia. Genome Res (2014) 24:1918–31. doi: 10.1101/gr.171645.113 PMC4248310 25224068

[53] Takaba H Morishita Y Tomofuji Y Danks L Nitta T Komatsu N. Fezf2 Orchestrates a Thymic Program of Self-Antigen Expression for Immune Tolerance. Cell (2015) 163:975–87. doi: 10.1016/j.cell.2015.10.013 26544942

[54] Wells KL Miller CN Gschwind AR Wei W Phipps JD Anderson MS. Combined Transient Ablation and Single-Cell RNA-Sequencing Reveals the Development of Medullary Thymic Epithelial Cells. Elife (2020) 9. doi: 10.7554/eLife.60188 PMC7771965 33226342

[55] Lopes N Boucherit N Santamaria JC Provin N Charaix J Ferrier P. Thymocytes Trigger Self-Antigen-Controlling Pathways in Immature Medullary Thymic Epithelial Stages. Elife (2022) 11. doi: 10.7554/eLife.69982 PMC8860447 35188458

[56] Koble C Kyewski B. The Thymic Medulla: A Unique Microenvironment for Intercellular Self-Antigen Transfer. J Exp Med (2009) 206:1505–13. doi: 10.1084/jem.20082449 PMC2715082 19564355

[57] Millet V Naquet P Guinamard RR. Intercellular MHC Transfer Between Thymic Epithelial and Dendritic Cells. Eur J Immunol (2008) 38:1257–63. doi: 10.1002/eji.200737982 18412162

[58] Yap JY Wirasinha RC Chan A Howard DR Goodnow CC Daley SR. Indirect Presentation in the Thymus Limits Naive and Regulatory T-Cell Differentiation by Promoting Deletion of Self-Reactive Thymocytes. Immunology (2018) 154:522–32. doi: 10.1111/imm.12904 PMC6002238 29411880

[59] Perry JS Lio CW Kau AL Nutsch K Yang Z Gordon JI. Distinct Contributions of Aire and Antigen-Presenting-Cell Subsets to the Generation of Self-Tolerance in the Thymus. Immunity (2014) 41:414–26. doi: 10.1016/j.immuni.2014.08.007 PMC4175925 25220213

[60] Klein L Kyewski B Allen PM Hogquist KA. Positive and Negative Selection of the T Cell Repertoire: What Thymocytes See (and Don't See). Nat Rev Immunol (2014) 14:377–91. doi: 10.1038/nri3667 PMC4757912 24830344

[61] Dhalla F Baran-Gale J Maio S Chappell L Hollander GA Ponting CP. Biologically Indeterminate Yet Ordered Promiscuous Gene Expression in Single Medullary Thymic Epithelial Cells. EMBO J (2020) 39:e101828. doi: 10.15252/embj.2019101828 31657037 PMC6939203

[62] Pinto S Michel C Schmidt-Glenewinkel H Harder N Rohr K Wild S. Overlapping Gene Coexpression Patterns in Human Medullary Thymic Epithelial Cells Generate Self-Antigen Diversity. Proc Natl Acad Sci USA (2013) 110:E3497–505. doi: 10.1073/pnas.1308311110 PMC3773787 23980163

[63] Brennecke P Reyes A Pinto S Rattay K Nguyen M Kuchler R. Single-Cell Transcriptome Analysis Reveals Coordinated Ectopic Gene-Expression Patterns in Medullary Thymic Epithelial Cells. Nat Immunol (2015) 16:933–41. doi: 10.1038/ni.3246 PMC4675844 26237553

[64] Tykocinski LO Sinemus A Rezavandy E Weiland Y Baddeley D Cremer C. Epigenetic Regulation of Promiscuous Gene Expression in Thymic Medullary Epithelial Cells. Proc Natl Acad Sci USA (2010) 107:19426–31. doi: 10.1073/pnas.1009265107 PMC2984162 20966351

[65] Meredith M Zemmour D Mathis D Benoist C. Aire Controls Gene Expression in the Thymic Epithelium With Ordered Stochasticity. Nat Immunol (2015) 16:942–49. doi: 10.1038/ni.3247 PMC4632529 26237550

[66] Irla M Guerri L Guenot J Serge A Lantz O Liston A. Antigen Recognition by Autoreactive CD4^+^ Thymocytes Drives Homeostasis of the Thymic Medulla. PloS One (2012) 7:e52591. doi: 10.1371/journal.pone.0052591 23300712 PMC3531460

[67] Irla M Hugues S Gill J Nitta T Hikosaka Y Williams IR. Autoantigen-Specific Interactions With CD4^+^ Thymocytes Control Mature Medullary Thymic Epithelial Cell Cellularity. Immunity (2008) 29:451–63. doi: 10.1016/j.immuni.2008.08.007 18799151

[68] Boehm T Scheu S Pfeffer K Bleul CC. Thymic Medullary Epithelial Cell Differentiation, Thymocyte Emigration, and the Control of Autoimmunity Require Lympho-Epithelial Cross Talk *via* Ltβr. J Exp Med (2003) 198:757–69. doi: 10.1084/jem.20030794 PMC2194183 12953095

[69] Akiyama T Shimo Y Yanai H Qin J Ohshima D Maruyama Y. The Tumor Necrosis Factor Family Receptors RANK and CD40 Cooperatively Establish the Thymic Medullary Microenvironment and Self-Tolerance. Immunity (2008) 29:423–37. doi: 10.1016/j.immuni.2008.06.015 18799149

[70] Metzger TC Khan IS Gardner JM Mouchess ML Johannes KP Krawisz AK. Lineage Tracing and Cell Ablation Identify a Post-Aire-Expressing Thymic Epithelial Cell Population. Cell Rep (2013) 5:166–79. doi: 10.1016/j.celrep.2013.08.038 PMC3820422 24095736

[71] White AJ Jenkinson WE Cowan JE Parnell SM Bacon A Jones ND. An Essential Role for Medullary Thymic Epithelial Cells During the Intrathymic Development of Invariant NKT Cells. J Immunol (2014) 192:2659–66. doi: 10.4049/jimmunol.1303057 PMC3948113 24510964

[72] Oh J Wu N Barczak AJ Barbeau R Erle DJ Shin JS. CD40 Mediates Maturation of Thymic Dendritic Cells Driven by Self-Reactive CD4^+^ Thymocytes and Supports Development of Natural Regulatory T Cells. J Immunol (2018) 200:1399–412. doi: 10.4049/jimmunol.1700768 PMC5809249 29321275

[73] Owen DL Mahmud SA Vang KB Kelly RM Blazar BR Smith KA. Identification of Cellular Sources of IL-2 Needed for Regulatory T Cell Development and Homeostasis. J Immunol (2018) 200:3926–33. doi: 10.4049/jimmunol.1800097 PMC5988981 29728511

[74] Hemmers S Schizas M Azizi E Dikiy S Zhong Y Feng Y. IL-2 Production by Self-Reactive CD4 Thymocytes Scales Regulatory T Cell Generation in the Thymus. J Exp Med (2019) 216:2466–78. doi: 10.1084/jem.20190993 PMC6829602 31434685

[75] Hu DY Wirasinha RC Goodnow CC Daley SR. IL-2 Prevents Deletion of Developing T-Regulatory Cells in the Thymus. Cell Death Differ (2017) 24:1007–16. doi: 10.1038/cdd.2017.38 PMC5442470 28362433

[76] Lio CW Hsieh CS. A Two-Step Process for Thymic Regulatory T Cell Development. Immunity (2008) 28:100–11. doi: 10.1016/j.immuni.2007.11.021 PMC2248212 18199417

[77] Burchill MA Yang J Vang KB Moon JJ Chu HH Lio CW. Linked T Cell Receptor and Cytokine Signaling Govern the Development of the Regulatory T Cell Repertoire. Immunity (2008) 28:112–21. doi: 10.1016/j.immuni.2007.11.022 PMC2430111 18199418

[78] Lee HM Bautista JL Scott-Browne J Mohan JF Hsieh CS. A Broad Range of Self-Reactivity Drives Thymic Regulatory T Cell Selection to Limit Responses to Self. Immunity (2012) 37:475–86. doi: 10.1016/j.immuni.2012.07.009 PMC3456990 22921379

[79] Leung MW Shen S Lafaille JJ. TCR-Dependent Differentiation of Thymic Foxp3^+^ Cells is Limited to Small Clonal Sizes. J Exp Med (2009) 206:2121–30. doi: 10.1084/jem.20091033 PMC2757883 19737865

[80] Lucca LE Axisa PP Aloulou M Perals C Ramadan A Rufas P. Myelin Oligodendrocyte Glycoprotein Induces Incomplete Tolerance of CD4^+^ T Cells Specific for Both a Myelin and a Neuronal Self-Antigen in Mice. Eur J Immunol (2016) 46:2247–59. doi: 10.1002/eji.201646416 27334749

[81] Kieback E Hilgenberg E Stervbo U Lampropoulou V Shen P Bunse M. Thymus-Derived Regulatory T Cells Are Positively Selected on Natural Self-Antigen Through Cognate Interactions of High Functional Avidity. Immunity (2016) 44:1114–26. doi: 10.1016/j.immuni.2016.04.018 27192577

[82] Fazilleau N Delarasse C Sweenie CH Anderton SM Fillatreau S Lemonnier FA. Persistence of Autoreactive Myelin Oligodendrocyte Glycoprotein (MOG)-Specific T Cell Repertoires in MOG-Expressing Mice. Eur J Immunol (2006) 36:533–43. doi: 10.1002/eji.200535021 16506290

[83] Hassler T Urmann E Teschner S Federle C Dileepan T Schober K. Inventories of Naive and Tolerant Mouse CD4 T Cell Repertoires Reveal a Hierarchy of Deleted and Diverted T Cell Receptors. Proc Natl Acad Sci USA (2019) 116:18537–43. doi: 10.1073/pnas.1907615116 PMC6744931 31451631

[84] Kalekar LA Schmiel SE Nandiwada SL Lam WY Barsness LO Zhang N. CD4^+^ T Cell Anergy Prevents Autoimmunity and Generates Regulatory T Cell Precursors. Nat Immunol (2016) 17:304–14. doi: 10.1038/ni.3331 PMC4755884 26829766

[85] Waldmann H Cobbold S. How do Monoclonal Antibodies Induce Tolerance? A Role for Infectious Tolerance? Annu Rev Immunol (1998) 16:619–44. doi: 10.1146/annurev.immunol.16.1.619 9597144

[86] Picca CC Simons DM Oh S Aitken M Perng OA Mergenthaler C. CD4+ CD25+ Foxp3+ Regulatory T Cell Formation Requires More Specific Recognition of a Self-Peptide Than Thymocyte Deletion. Proc Natl Acad Sci (2011) 108:14890–95. doi: 10.1073/pnas.1103810108 PMC3169143 21873239

[87] Wirasinha RC Davies AR Srivastava M Sheridan JM Sng XYX Delmonte OM. *Nfkb2* Variants Reveal a P100-Degradation Threshold That Defines Autoimmune Susceptibility. J Exp Med (2021) 218 :18537-43. doi: 10.1084/jem.20200476 PMC7595743 33107914

[88] Zammit NW Siggs OM Gray PE Horikawa K Langley DB Walters SN. Denisovan, Modern Human and Mouse *TNFAIP3* Alleles Tune A20 Phosphorylation and Immunity. Nat Immunol (2019) 20:1299. doi: 10.1038/s41590-019-0492-0 31534238

[89] Fulford TS Grumont R Wirasinha RC Ellis D Barugahare A Turner SJ. C-Rel Employs Multiple Mechanisms to Promote the Thymic Development and Peripheral Function of Regulatory T Cells in Mice. Eur J Immunol (2021) 51:2006–26. doi: 10.1002/eji.202048900 33960413

[90] Randall KL Law HD Ziolkowski AF Wirasinha RC Goodnow CC Daley SR. DOCK8 Deficiency Diminishes Thymic T-Regulatory Cell Development But Not Thymic Deletion. Clin Transl Immunol (2021) 10:e1236. doi: 10.1002/cti2.1236 PMC7790591 33437483

[91] Tai X Erman B Alag A Mu J Kimura M Katz G. Foxp3 Transcription Factor is Proapoptotic and Lethal to Developing Regulatory T Cells Unless Counterbalanced by Cytokine Survival Signals. Immunity (2013) 38:1116–28. doi: 10.1016/j.immuni.2013.02.022 PMC3700677 23746651

[92] Owen DL Mahmud SA Sjaastad LE Williams JB Spanier JA Simeonov DR. Thymic Regulatory T Cells Arise *via* Two Distinct Developmental Programs. Nat Immunol (2019) 20:195–205. doi: 10.1038/s41590-018-0289-6 30643267 PMC6650268

[93] McCaughtry TM Wilken MS Hogquist KA. Thymic Emigration Revisited. J Exp Med (2007) 204:2513–20. doi: 10.1084/jem.20070601 PMC2118501 17908937

[94] Thiault N Darrigues J Adoue V Gros M Binet B Perals C. Peripheral Regulatory T Lymphocytes Recirculating to the Thymus Suppress the Development of Their Precursors. Nat Immunol (2015) 16:628–34. doi: 10.1038/ni.3150 25939024

[95] Weist BM Kurd N Boussier J Chan SW Robey EA. Thymic Regulatory T Cell Niche Size is Dictated by Limiting IL-2 From Antigen-Bearing Dendritic Cells and Feedback Competition. Nat Immunol (2015) 16:635–41. doi: 10.1038/ni.3171 PMC4439282 25939026

[96] McCarthy NI Cowan JE Nakamura K Bacon A Baik S White AJ. Osteoprotegerin-Mediated Homeostasis of Rank^+^ Thymic Epithelial Cells Does Not Limit Foxp3^+^ Regulatory T Cell Development. J Immunol (2015) 195:2675–82. doi: 10.4049/jimmunol.1501226 PMC4560491 26254339

[97] Cowan JE McCarthy NI Anderson G. CCR7 Controls Thymus Recirculation, But Not Production and Emigration, of Foxp3^+^ T Cells. Cell Rep (2016) 14:1041–48. doi: 10.1016/j.celrep.2016.01.003 PMC4751304 26832402

[98] Invernizzi P Gershwin ME. The Genetics of Human Autoimmune Disease. J Autoimmun (2009) 33:290. doi: 10.1016/j.jaut.2009.07.008 19682858

[99] Dendrou CA Petersen J Rossjohn J Fugger L. HLA Variation and Disease. Nat Rev Immunol (2018) 18:325–39. doi: 10.1038/nri.2017.143 29292391

[100] Lenz TL Deutsch AJ Han B Hu X Okada Y Eyre S. Widespread Non-Additive and Interaction Effects Within HLA Loci Modulate the Risk of Autoimmune Diseases. Nat Genet (2015) 47:1085–90. doi: 10.1038/ng.3379 PMC4552599 26258845

